# Enhancement of Pathogen Toxicity by Feeding *Reticulitermes chinensis* Snyder Sonicated Bacteria Expressing Double-Stranded RNA That Interferes with Olfaction

**DOI:** 10.3390/insects14020140

**Published:** 2023-01-30

**Authors:** Dabao Jiang, Xiaoyu Lu, Ling Zhang, Fang Tang

**Affiliations:** 1 Co-Innovation Center for Sustainable Forestry in Southern China, Nanjing Forestry University, Nanjing 210037, China; 2 College of Forestry, Nanjing Forestry University, Nanjing 210037, China

**Keywords:** termite, Orco, RNA interference, biocontrol, pathogen

## Abstract

**Simple Summary:**

*Reticulitermes chinensis* Snyder is an important pest that destroys trees and buildings and causes great damage. The present study investigated the role of *RcOrco*. A method for preparing large amounts of dsRNA using sonication of engineered bacteria was established. The dsRNA produced using this method was ingested by *R. chinensis* via feeding, which decreased the expression level of *RcOrco* and survival rate. The results of this study enhance our knowledge of *orco* function in social insects and propose new potential targets for termite control.

**Abstract:**

*Reticulitermes chinensis* Snyder is a serious pest in China, and the odorant receptor co-receptor gene *RcOrco* plays a crucial role in olfaction. However, the function of *RcOrco* in the resistance of termites to entomopathogens has not been reported. We constructed ds*RcOrco*-HT115 engineered bacteria based on the *RcOrco* sequence from the full-length transcriptome data of *R. chinensis*. The engineered bacteria expressed dsRNA of *RcOrco*. Sonication was used to inactivate the dsRNA-HT115 strain and obtain a large amount of ds*RcOrco*. The ds*RcOrco* produced using this method overcame the problem that genetically engineered bacteria could not be applied directly and improved its effectiveness against termites. Bioassays using the ds*RcOrco* generated using this method showed that ds*RcOrco* significantly increased the toxicity of the bacterial and fungal pathogens to *R. chinensis*. The present study showed, for the first time, the function of *Orco* in termite resistance to pathogens, and the results provide a theoretical basis for the development and application of termite RNA biopesticides.

## 1. Introduction

Insects use their olfactory system to recognize various odorants in their environment to find food, mates, and avoid natural enemies [1]. Odors are primarily detected by odorant receptors (ORs) expressed on olfactory receptor neurons (ORNs) that project to the central nervous system [2]. ORs are essential in olfaction and are divided into two subclasses, the traditional OR and odorant receptor co-receptor (Orco) [3]. ORs convert chemical stimuli into electrical signals [4] to play an important role in the odor recognition process. Orco and ORs form an Orco-OR heterotetramer that constitutes a ligand-gated ion channel [5]. Orco is highly conserved and plays an essential role in insect olfaction. Traditional ORs cannot function alone in the absence of Orco, and the absence of Orco impairs olfaction and olfactory behavior. For example, the knockout of Orco reduced olfactory sensitivity and affected insect behavior and physiology [6,7]. Orco mutations in mosquitoes, *Protaetia brevitarsis* (Lewis)*,* resulted in an inability to find hosts and impaired responses to odorants such as sex pheromones and volatiles [8,9]. *Blattella germanica* (L.) responded slowly to odorants from food after *Orco* was silenced [10]. *Orco* mutation ants exhibited a behavioral phenotype with loss of the olfactory system and reduced response to odorants [11]. Termite workers have limited visual capabilities and communicate with nest mates primarily via olfactory channels to convey pheromonal and other chemical cues. Knockout of the *Orco* gene affects the ability of *Odontotermes formosanus* (Shiraki) to discriminate between nestmates, which increased aggression toward nest mates [12]. Previous studies on the functions of *Orco* have mostly focused on foraging, mating, and host selection. However, whether *Orco* affects insect resistance to pathogens is not clear.

RNAi is a widely used tool to study gene function, and it has shown great potential in developing new pest management strategies [13]. RNAi is a sequence-specific gene silencing phenomenon induced by double-stranded RNA (dsRNA) at the post-transcriptional level, and it has been used to study different species of insects, including Lepidoptera, Hymenoptera, and Diptera [14]. It is feasible to research the function of termite genes by feeding or injecting synthetic dsRNA [15,16,17]. DsRNA is considered a new generation of RNA biopesticides with great potential for biological control. For example, feeding *Spodoptera littoralis* (Boisduval) larvae *sl 102* dsRNA enhanced the toxicity of Bt [18]. The use of specific dsRNA feeding to silence *termicin* and *GNBP* genes in *Reticulitermes flavipes* (Kollar) reduced the survival of termites infected with pathogens [19]. Silencing of the β-1,3-glucan binding protein gene effectively increased the susceptibility of *Plutella xylostella* (L.) to entomopathogenic fungi [20]. The dsRNA or siRNA used in these studies was prepared directly by in vitro synthesis or by extracting the expressed dsRNA from the engineered bacteria using chemical reagents (e.g., TRIzol or phenol) [21]. These methods are expensive or time-consuming, which is not conducive to large-scale application in dsRNA production.

*Reticulitermes chinensis* Snyder is a serious pest that damages forests and buildings. It is widely distributed in China and causes huge economic losses annually [22]. Although some researchers hope to only use pathogenic bacteria to control *R. chinensis,* Chouvenc et al. have demonstrated that it is not feasible to control termites by using microorganisms alone. Instead, by focusing research on understanding the complex biology of termites, particularly their various defense mechanisms, investigators may find a way for pathogens to bypass such mechanisms, and improve prospects for biological control [23]. Therefore, the combination of pathogenic bacteria and RNAi is the development direction of termite biological control. The silencing of *Orco* in *R. chinensis* impaired their olfaction. However, whether *Orco* silencing in *R. chinensis* affects their responses to pathogen infections is not known. The present study constructed dsRNA-HT115 bacteria and sonicated them to produce a large amount of ds*RcOrco* for direct application. We used the ds*RcOrco* produced by this method to investigate whether *Orco* played an important role in the defensive responses of *R. chinensis* to entomopathogen infection. The present study provides a basis for the development and application of termite RNA biopesticides. Furthermore, the results provide a theoretical basis for the association of RNAi of termites with pathogens.

## 2. Materials and Methods

### 2.1. Insects and Microorganisms

The *R. chinensis* termites used in this study were collected from six colonies in Jiangsu Province, China, and were maintained separately in plastic containers (20 cm × 15 cm × 15 cm). The colonies were maintained at 25 ± 1 °C, 90% ± 5% relative humidity, and 24 h darkness. Only healthy workers were selected for the experiment.

Ten microliters of preserved *Serratia marcescens* Bizio (SM1) were dropped into 50 mL of fermentation medium and incubated at 30 °C and 180 rpm for 48 h. *Bacillus thuringiensis* Berliner (Bt) was cultured in LB medium at 37 °C and 180 rpm for 1 week. *Beauveria bassiana* (Bals.-Criv.) Vuill. (Bb) was incubated in a PDA medium for 2 weeks at 30 °C. A spore suspension was prepared by rinsing *B. bassiana* spores in the medium using 0.1% Tween 80.

### 2.2. Synthesis of dsRNA

The *RcOrco* gene sequence (1418bp) was obtained from the full-length transcriptome data of *R. chinensis* (Table S1). Total RNA was extracted from 100 mg of termite worker antennae using TaKaRa RNAiso Plus (TRIzol) according to the manufacturer’s protocol. The extracted total RNA was reverse transcribed to cDNA using the Primescript^TM^ 1st Strand cDNA Synthesis Kit (Takara, Dalian, Liaoning, China). Based on the ORF of the *RcOrco* gene, a pair of specific amplification primers (Table S2) was designed by predicting the possible RNAi sites using the online website (http://sidirect2.RNAi.jp/ accessed on 20 December 2022), and PCR amplification was performed with the designed primers. The PCR products were identified using 1% agarose gel electrophoresis, and the target fragments were recovered using a Gel Extraction Kit. The PCR product was ligated to the L4440 vector via double digestion and ligation with T4 DNA ligase. The ligated vector was transformed into HT115-competent cells. After sequencing was confirmed, the transformed HT115 bacteria were cultured at 37 °C overnight and stored in 25% bacterial fluid + 75% glycerol at −80 °C and recorded as ds*RcOrco*-HT115. *GFP* was used as a control for the same methodology as above, and the dsGFP-producing strain was recorded as ds*GFP*-HT115.

### 2.3. Inactivation of dsRNA-HT115 by Sonication for dsRNA Preparation

Ten microliters of preserved ds*RcOrco*-HT115 bacteria were added to 50 mL of liquid Luria–Bertani (LB) broth, and ampicillin and tetracycline were added to final concentrations of 75 μg/mL and 12.5 μg/mL, respectively. The bacterial solution was cultured at 37 °C for 24 h with shaking (180 rpm). One milliliter of the bacterial solution was added to 50 mL of LB liquid medium, and the concentrations of ampicillin and tetracycline in the medium were the same as listed above. The solutions were incubated with shaking at 37 °C until the OD600 = 0.4–0.6, then 400 μL isopropyl-beta-D -thiogalactopyranoside (IPTG, a final concentration of 0.8 mM) was added to induce the expression of dsRNA. The bacteria were incubated at 37 °C with shaking for 4 h. Sonication treatment was used to kill the dsRNA-HT115 bacteria, and disrupting the cell wall and cell membrane of the bacteria promoted the release of dsRNA. Eight milliliters of ds*RcOrco*-HT115 bacteria were centrifuged at 12,000 rpm for 5 min, the supernatant was removed, and 5 mL of DEPC water was added and mixed well. The bacterial suspension was crushed using an ultrasound homogenizer with 50% power, 3 s on and 6 s off. One hundred microliters of the bacterial solution were aspirated every 20 min, added to LB medium (containing 100 μg/mL ampicillin and 12.5 μg/mL tetracycline), and observed after 24 h.

### 2.4. Establishment of the Standard Curve for dsRcOrco

IPTG was added to the dsRNA-HT115 bacterial solution at a final concentration of 0.8 mM. The bacterial solution was incubated at 37 °C for 4 h and centrifuged for 5 min (8500 rpm). The supernatant was removed, and dsRNA was extracted using TRIzol. The concentration of dsRNA was expressed by measuring the UV absorbance of the dsRNA solution at 260 nm. Real-time fluorescent quantitative PCR (qRT-PCR) was performed by diluting the starting solution containing 1000 ng/μL dsRNA in four gradients at a 10-fold dilution as a template, and primers were designed for qRT-PCR (Table S2), and the CT values obtained by qRT-PCR were plotted against the concentration of dsRNA to establish the standard curve of dsRNA. Using the absolute quantification method, the concentration of dsRNA in the inactivated solution was determined by correlating the CT values with the established standard curve to ensure the consistency of the concentration of dsRNA in the inactivated solution in RNAi experiments [24].

### 2.5. Interference Efficiency of dsRcOrco

Twenty worker termites were put into Petri dishes (7 cm) lined with dry filter paper, which was treated with 400 μL of ds*RcOrco* (1.5 μg/μL dsRNA) and Nile blue (1% *w*/*v*). ds*GFP* (1.5 μg/μL dsRNA) and Nile bule (1% *w*/*v*) were used as positive controls, and DEPC water and Nile blue (1% *w*/*v*) were used as negative controls. The termites were placed in darkness. The total RNA of termite workers collected at different treatment times was extracted using TRIzol, and primers were designed outside the dsRNA region (Table S2). The expression levels of the *RcOrco* gene of termites at different treatment times (6, 12, 24, and 48 h) were detected using qRT-PCR, and *β-actin* and *HSP 70* were used as internal reference genes (Table S2). The qRT-PCR data were calculated using the 2^-ΔΔCT^ method [25,26].

### 2.6. Bioassay of dsRNA Combined with Pathogens to R. chinensis

A number of *R. chinensis* workers for this experiment were placed into Petri dishes with dry filter paper containing 400 μL of ds*RcOrco* (1.5 μg/μL dsRNA) and Nile blue (1% *w*/*v*) (ds*RcOrco* treatment), DEPC water and Nile blue (1% *w*/*v*) (CK treatment), ds*GFP* (1.5 μg/μL dsRNA) and Nile blue (1% *w*/*v*) (ds*GFP* treatment) for 12 h. The workers from the CK*,* ds*GFP,* and ds*RcOrco* treatments were divided into two groups (uninfected and bacterial infection group), respectively. Two microliters of culture medium were placed on each worker’s pronotum [uninfected groups: CK (negative control), ds*GFP* (positive control), and ds*RcOrco*]; 2 μL of Bt (3.3 × 10^9^ cells/mL) was applied to the pronotum of each worker termite that was dyed blue (bacterial infection groups: CK-Bt, ds*GFP*-Bt, and ds*RcOrco*-Bt). Three replicates were from three different colonies and recorded every 24 h. In addition, the processing and grouping method of 2 μL of SM1 (3.8 × 10^11^ cells/mL) or 2 μL of Bb spore suspension (4.9 × 10^9^ cells/mL) was the same as Bt.

### 2.7. Statistical Analysis

InStat software (GraphPad, San Diego, CA, USA) was used to analyze the collected data. Two-tailed unpaired Student’s *t*-test was used to compare differences between the two samples. The statistical significance of multiple sample comparisons was determined using one-way ANOVA and Tukey’s HSD test. A *p*-value < 0.05 indicated a significant difference. RNAi bioassay data were analyzed using the log-rank Mantel-Cox test (GraphPad Prism 8.0.2, GraphPad Software, San Diego, CA, USA).

## 3. Results

### 3.1. Construction of Inactivated dsRNA-HT115 System for dsRNA Preparation

#### 3.1.1. Inactivation of *dsRcOrco*-HT115

**Figure 1:**
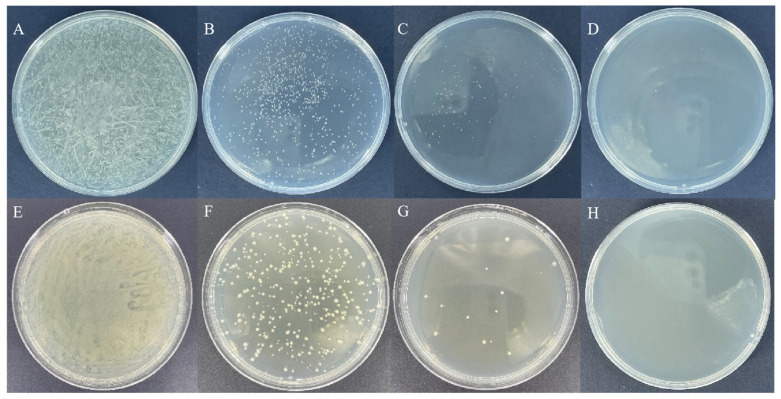
Growth condition of ds*RcOrco*-HT115 on medium after sonication. ds*RcOrco*-HT115 (**A**–**D**) (**A**): 0 min. (**B**): 20 min. (**C**): 40 min. (**D**): 60 min. ds*GFP*-HT115 (**E**–**H**) (**E**): 0 min. (**F**): 20 min. (**G**): 40 min. (**H**): 60 min.

The bacterial solution was sonicated using an ultrasound homogenizer, and the samples were collected every 20 min. After ultrasonic treatment, the bacterial solution was plated in Petri dishes containing LB medium. The results showed that the number of colonies continued to decrease as the sonication time increased, and the Petri dishes no longer showed colonies after 1 h of sonication (Figure 1). Therefore, we used 1 h of sonication for subsequent experiments.

#### 3.1.2. The Standard Curve for dsRcOrco

**Figure 2:**
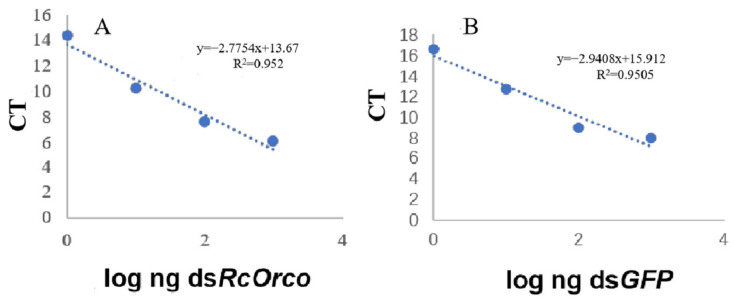
Standard curves of dsRNA (**A**) *dsRcOrco* and (**B**) *dsGFP*.

The logarithms of the four gradient concentrations were plotted against the corresponding CT values. The standard curve for ds*RcOrco* was calculated as y = −2.7754x + 13. 67 (R^2^ = 0.952) (Figure 2A), and the standard curve for ds*GFP* was established in the same manner as y = −2.9408 x + 15. 912 (R^2^ = 0.9505) (Figure 2B).

#### 3.1.3. Interference Efficiency of *dsRcOrco*

**Figure 3:**
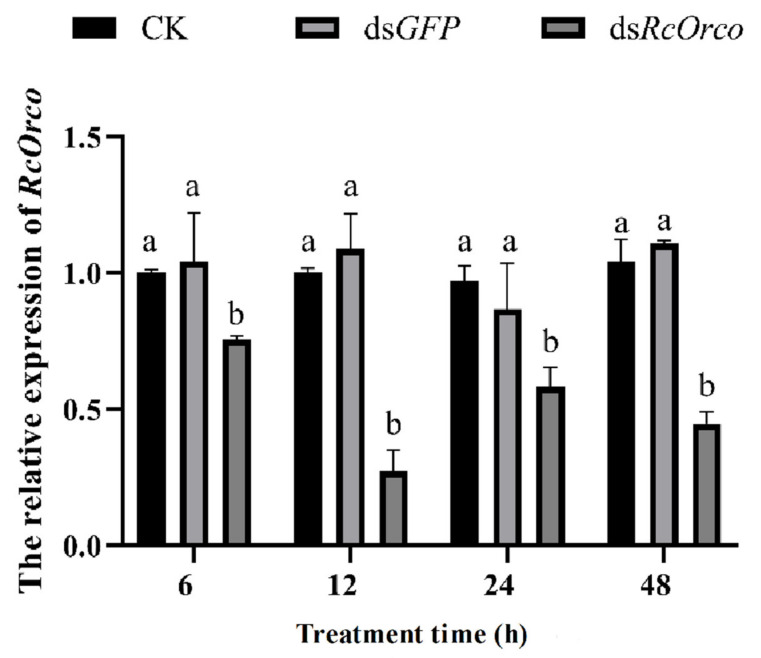
The effect of *dsRcOrco* on the relative expression of *RcOrco* in R. chinensis. The letters above the columns indicate different expressive differences.

After ds*RcOrco* treatment, qRT-PCR was used to quantify the expression levels of the *RcOrco* gene at different treatment times. The results showed that the expression of *RcOrco* was suppressed at all time periods, and the transcript levels of the *RcOrco* gene decreased by 24.5%, 72.7%, 41.6%, and 55.5% at 6, 12, 24, and 48 h, respectively, with the greatest decrease in the transcript levels of the *RcOrco* gene at 12 h (Figure 3).

### 3.2. Effects of Three Pathogens on dsRNA-Treated R. chinensis

#### 3.2.1. Bioassay of *dsRcOrco* Combined with Bt

**Figure 4:**
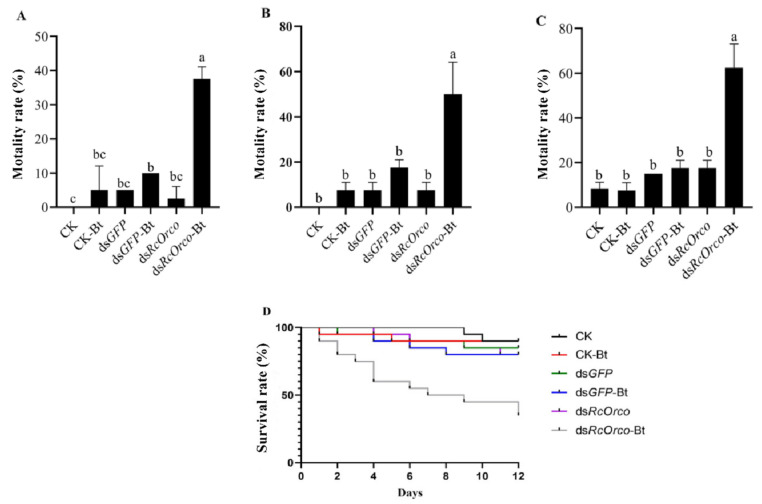
Bioassay of *dsRcOrco* combined with Bt in *R. chinensis*. (**A**) Mortality rate of each treatment group at 4 d. (**B**) Mortality rate of each treatment group at 8 d. (**C**) Mortality rate of each treatment group at 12 d. (**D**) Survival rate of each treatment group. The letters above the columns indicate different expressive differences.

The results of using Bt to treat termites after silencing *RcOrco* showed that the mortality rate of the ds*RcOrco*-Bt group (37.5%) was significantly higher (F = 46.2; df = 5.18; *p* < 0.0001) than the other treatment groups at 4 d (Figure 4A). The mortality rate of the ds*RcOrco*-Bt group (50%) was significantly higher (F = 23.4; df = 5.18; *p* < 0.0001) than the other treatment groups at 8 d (Figure 4B). The ds*RcOrco*-Bt group reached the highest mortality rate of 62.5% at 12 d, which was significantly higher (F = 48.305; df = 5.18; *p* < 0.0001) than the CK-Bt group (7.5%) and ds*GFP*-Bt group (17.5%) (Figure 4C). The results of the survival curves showed that the survival rate of the ds*RcOrco*-Bt group was lower than the other Bt treatment groups (CK-Bt group and ds*GFP*-Bt group), and the survival rate of the Bt treatment groups (CK-Bt, ds*GFP*-Bt, and ds*RcOrco*-Bt) was lower than other groups (CK group, ds*GFP* group, and ds*RcOrco* group) (Figure 4D).

#### 3.2.2. Bioassay of *dsRcOrco* Combined with SM1

**Figure 5:**
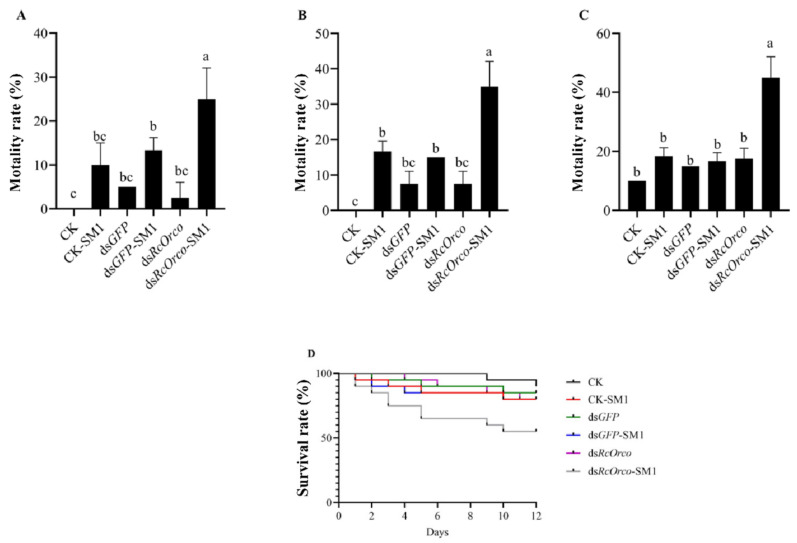
Bioassay of *dsRcOrco* combined with SM1 in *R. chinensis* (**A**) Mortality rate of each treatment group at 4 d. (**B**) Mortality rate of each treatment group at 8 d. (**C**) Mortality rate of each treatment group at 12 d. (**D**) Survival rate of each treatment group. The letters above the columns indicate different expressive differences.

The results of using SM1 to treat termites after silencing *RcOrco* showed that the mortality rate of ds*RcOrco*-SM1 (F = 15.57; df = 5,18; *p* < 0.0001) was the highest among all treatment groups, reaching 25% at 4 d (Figure 5A). The mortality rate of the ds*RcOrco*-SM1 group was 35% at 8 d, which was 18.34% and 20% higher (F = 31.48; df = 5,18; *p* < 0.0001) than the CK-SM1 and ds*GFP*-SM groups, respectively (Figure 5B). The mortality rate of the ds*RcOrco*-SM1 treatment group at 12 d (F = 34.974; df = 5,18; *p* < 0.0001) was 26.67% and 28.33% higher than the CK-SM1 and ds*GFP*-SM1 groups, respectively (Figure 5C). The results of the survival curves showed that the survival rate of the ds*RcOrco*-SM1 group was lower than the other SM1 treatment groups (CK-SM1 group and ds*GFP*-SM1 group), and the survival rate of the SM1 treatment groups (CK-SM1, ds*GFP*-SM1, and ds*RcOrco*-SM1) was lower than other groups (CK group, ds*GFP* group, and ds*RcOrco* group) (Figure 5D).

#### 3.2.3. Bioassay of *dsRcOrco* Combined with Bb

**Figure 6:**
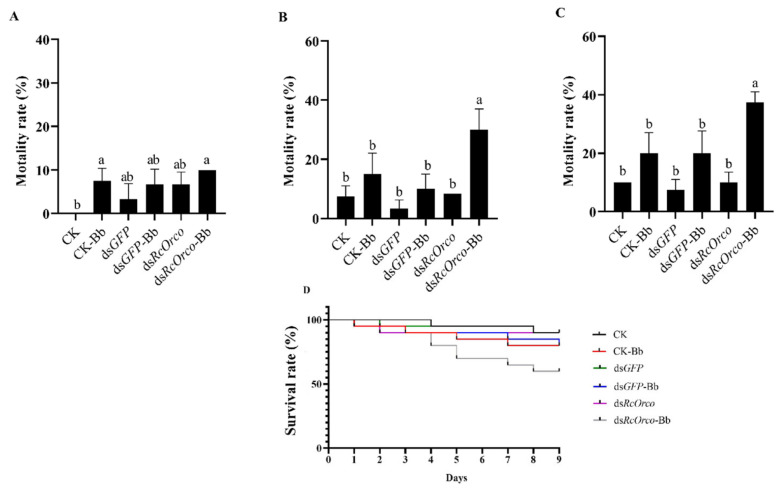
Bioassay of *dsRcOrco* combined with Bb in *R. chinensis*. (**A**) Mortality rate of each treatment group at 3 d. (**B**) Mortality rate of each treatment group at 6 d. (**C**) Mortality rate of each treatment group at 9 d. (**D**) Survival rate of each treatment group. The letters above the columns indicate different expressive differences.

The results of using Bb to treat termites after silencing *RcOrco* showed that the mortality rate of the ds*RcOrco*-Bb group was 30% at 6 d (F = 10.986; df = 5,18; *p* = 0.0004), which was significantly higher than the CK-Bb group (15%) and the ds*GFP*-Bb group (10%) (Figure 6B), and the mortality rate of the ds*RcOrco*-Bb group was the highest at 37.5% at 9 d (F = 15.429; df = 5,18; *p* < 0.0001), which was higher than the ds*GFP*-Bb group and the CK-Bb group (Figure 6C). Survival curves showed that the survival rate of the ds*RcOrco*-Bb group was lower than the other Bb treatment groups (CK-Bb group and ds*GFP*-Bb group), and the survival rate of the Bb treatment groups (CK-Bb, ds*GFP*-Bb, and ds*RcOrco*-Bb) was lower than other groups (CK group, ds*GFP* group, and ds*RcOrco* group) (Figure 6D).

## 4. Discussion

One common approach to silence the expression of target genes in insect RNAi experiments is the injection of dsRNA or siRNA, but the use of microinjection may lead to higher mortality of small insects, such as termites. This methodology also requires additional equipment and techniques to perform, compared to the feeding method which requires a larger amount of dsRNA [27]. There are limitations in the application of engineered bacteria due to environmental safety concerns. Therefore, the engineered bacteria dsRNA-HT115 expressing dsRNA cannot be used directly, and feeding *Spodoptera exigua* (Hübner) dsRNA-engineered bacteria failed to improve insecticidal activity [28], which may be due to the continuous slow release of dsRNA after the engineered bacteria enter the insects and the low concentration of dsRNA that cannot effectively trigger RNAi [29]. However, sonication disrupts the cell wall and cell membrane of engineered bacteria to cause the release of dsRNA from the bacteria. The dsRNA that is released after sonication pretreatment of engineered bacteria significantly increased the mortality of *Maruca vitrata* (Fabricius) larvae [30]. The use of inactivated engineered bacteria to produce dsRNA has not been reported in termites. Our study used sonication-inactivated bacteria for RNAi experiments, which allowed for the rapid preparation of dsRNA in large quantities and provided technical support for the development of novel dsRNA biopesticides for termites and the use of inactivated bacteria expressing dsRNA to ensure environmental safety. Therefore, this method has broad application in pest control.

Social insects, such as bees, have a lower number of immune genes than solitary insects, such as mosquitoes and flies [31]. Despite these obvious weaknesses, social insects, such as bees, ants, and termites, cope well with pathogens, and termite nests are rarely destroyed by pathogenic microbial infections, which may be related to social immune strategies to avoid pathogenic infection. However, the number of genes for odorant receptors and gustatory receptors is significantly higher in social insects than in solitary insects [32,33], which may be because social insects have evolved to strengthen social immunity by enhancing their olfactory ability to compensate for the deficiency of humoral and cellular immunity and enhance the ability of the group to resist diseases. Termites can detect the presence of *Metarhizium anisopliae* (Metsch.) Sorokin at a distance via olfaction and avoid physical contact with pathogenic fungi [34]. Termites show a marked aversion to the odor of specific volatiles of *Pseudomonas aeruginosa* (Schroeter) Migula. When infected workers enter the nest, other workers recognize them and remove spores from the body surface of the infected workers by grooming behavior [35,36]. The termites also use high-frequency shaking to warn nest mates not to approach the infected worker termites [37,38]. The basis of these behavioral defenses is that termites accurately identify the odor of pathogens and infected individuals via olfaction. Therefore, the silencing of a key gene in termite olfactory recognition, *Orco*, will affect the olfaction of termites and disrupt and reduce their social immunity to pathogen infection.

RNAi specifically suppresses gene expression and has great potential for pest control [39]. The use of RNAi in combination with entomopathogens is an important aspect of biological control. The innate and social immunity of termites makes them resilient to most pathogens, and the use of RNAi technology to impair this immunity in combination with entomopathogens may be effective for biological control. *Termicin* silencing in *O. formosanus* termites using RNAi significantly increased the toxicity of SM1 [40]. *Orco* functions in host finding, foraging, courtship, mating, pheromone recognition, and other behaviors [4,10,11,41]. Termites are social insects with strong social immunity, and olfaction plays an important role in the social immunity process. Therefore, we used *RcOrco* as a target gene for RNAi to investigate its function in the resistance of *R. chinensis* termites to pathogen infection. Our results showed that *RcOrco* silencing significantly reduced termite survival when *R. chinensis* was infected with pathogens. The combination of ds*RcOrco* and pathogens significantly enhanced virulence. We suspect that the silencing of *RcOrco* with ds*RcOrco* impaired the ability of termites to accurately recognize the odors of pathogens, which may reduce the frequency of grooming behavior to remove pathogens and weaken the social immunity of termites to allow the pathogenic microorganisms to remain on the termite cuticle and enter the hemocoel. These suggested mechanisms must be explored in future studies.

## 5. Conclusions

In summary, the results showed that feeding termites with ds*RcOrco* obtained from inactivated engineered bacteria silenced the expression of *Orco* in *R. chinensis*, and silencing of the *RcOrco* gene enhanced the virulence of Bt, SM1, and Bb. Therefore, *RcOrco* plays an important role in the immunity of *R. chinensis* to pathogen infection, and it should be considered a potential target for the development of RNAi agents to control pests.

## Data Availability

The data presented in this study are available on request from the corresponding author.

## Supplementary Materials

The following supporting information can be downloaded at: https://www.mdpi.com/article/10.3390/insects14020140/s1, Table S1: The sequence of RcOrco. Table S2: Primers used for this study.
