# Supplementary material for: Enhancement of Pathogen Toxicity by Feeding Reticulitermes chinensis Snyder Sonicated Bacteria Expressing Double-Stranded RNA That Interferes with Olfaction
Source: Insects. 2023 Jan 30;14(2):140. doi: 10.3390/insects14020140 (PMC9965219; doi:10.3390/insects14020140)
Supplement: Supplementary file 1 [file insects-14-00140-s001.zip › Table S1. The sequence of RcOrco.pdf]

Table S1. The sequence of *RcOrco*

ATGTACAAGTTCAGGTTACACGGTCTGGTCGCGGACTTGTGGCCGCTGAT
CAGAATCATGCAAATGACCGGCTTCTTTTTGCTGGACTATCACGAAGACA
TGAGCTTCGGCTGGACATCCATCAGGACCGGGTACGCTTCAACTGTGTCT
TGTTTCATGGTCATACAATACCTGCTCTTATTCATCAACCTCATGAAGCA
AGCCGAAGATGTCAATGACCTGACAGCCAACACGATCACCGTACTTTTCT
TCCTCCATTCGCTGATAAAATTTTTCTACTTTGCGATCCGTCGCGCCAAG
TTCTACCGAACTTTGGCAACATGGAACAACGCCAACAGCCATCCGTTGTT
TTCCGAAAACCAGTCGCGCCACCACGCTACGGCGGTCGGTAGCATGAGGC
GCCTGGTCATGAACGTGGGCATCGGCACCATCGTCAGCGGGTTTGCCTGG
ACCGCCATCACCTTCATCGGAGACAGTGTGCACGAAATACCCGACCCAGA
TAATGGCAATGAAACTATTTTCGAGGAGGTGCCGCGTCTCATGTTACGTT
CATGGTATCCCTGGAATGCTTTGTCTGGCGGGGGCTACGTCGTGTCCTTC
ATCATCCAGATATTGTGGCTGTTCCTGGCCCTGTCGCATGCCATGATGAT
GGACACGATGTTCTGCTGTTGGCTCATCTACACGTGTGAGCAACTCATTC
ATCTCAAAGAGATCATGAAGCCGCTAATGGAGCTCAGCGCATCGCTGGAC
ACTGTCGTGCCTCACTCTGCAGATCTGTTCCGTGCAGTCAGCGCCACCAC
CAACGCTCCCATAACTTCAGGTGACGGCGAGGGTATTCGAGCTATATACA
GCAACCAGCACGACTTCTCAAATTTCCGCCTGAATACCGGCACACTTGCC
AACGTCAACAGTGGCTCTGTTGGACCAAACGGACTGACAAAGAAACAGGA
ACTGCTGGTGCGGTCCGCCATCAAGTACTGGGTGGAGAGGCACAAACACG
TGGTCCGATTCGTCAGTAATATTGGAGACACATACGGCTCGGCACTGCTA
CTGCACATGCTAACTAGCACAGTGGCTTTGACACTGCTCGCCTACCAGGC
CACAAAGATTGACACCGTGAATGTGTATGCCTGCACAGTACTCGGCTATC
TAGTATACTCACTGGCCCAGGTGTTCCTCTTCTGCTTCTTTGGCAACCGT
CTCATCGAAGAGAGCTCGTCAGTGATGGAGGCTGCCTACAGCTGTCAGTG
GTATGACGGTTCGGAGGAAGCAAAAACTTTTATCCAGATCGTATGTCAAC
AATGTCAGAAAGCCATGAGCATTTCTGGAGCCAAATTCTTCACAGTGTCA
CTCGACTTGTTTGCTTCGGTGTTGGGTGCTATTGTGACCTACTTCATGGT
GCTGGTCCAGCTCAACTAG
