# Supplementary material for: Enhancement of Pathogen Toxicity by Feeding Reticulitermes chinensis Snyder Sonicated Bacteria Expressing Double-Stranded RNA That Interferes with Olfaction
Source: Insects. 2023 Jan 30;14(2):140. doi: 10.3390/insects14020140 (PMC9965219; doi:10.3390/insects14020140)
Supplement: Supplementary file 1 [file insects-14-00140-s001.zip › Table S2. Primers used for this study.pdf]

Table S2. Primers used for this study

Primer names	Forward Primer Sequences (5′-3′)	Reverse Primer Sequences (5′-3′)	Experiments
dsRcOrco	ATTT**GCGGCCGC**TGGACACTGTGGTGCCTAAC	CCC**AAGCTT**GATCACGGTGCAGGCATAGA	RNAi
dsGFP	ATC**GGAGCT**CTAGTTGAACGGATCCATCTTCA	CCC**AAGCTT**AGAACTTTTCACTGGA	
dsGFP	TCGAGTTTGTGTCCGAGAATG	CAAAGATGACGGGAACTACAAGA	Standard curve
dsRcOrco	GGAGACAGTGTGCACGAAATA	CCAGGGATACCATGAACGTAAC	
RcOrco	CTGTACCAGCACCATGAAGTAG	GAGCATCTCTGGAGCCAAAT	qRT-PCR
β-actin	GGCCTCACTGTCCACTTTCC	CTTGCTGTCGATGTGTGAACG	
HSP 70	CCAACTTTGCTGCAATGCGA	GCACCTGGCTTAGCACGTAT	

Note: The bold letters of primer are the restriction site; *β-actin* and *HSP 70* are the internal reference genes in this study.
